# On dispersion curve coloring for mechanical metafilters

**DOI:** 10.1038/s41598-022-23491-4

**Published:** 2022-11-21

**Authors:** Andrea Bacigalupo, Maria Laura De Bellis, Giorgio Gnecco, Federico Nutarelli

**Affiliations:** 1 DICCA – University of Genoa, Genoa, Italy; 2 INGEO – University of Chieti-Pescara, Chieti, Italy; 3 AXES – IMT School for Advanced Studies, Lucca, Italy; 4 ICRIOS – Bocconi University, Milan, Italy

**Keywords:** Computational methods, Structural materials

## Abstract

This paper formalizes smooth curve coloring (i.e., curve identification) in the presence of curve intersections as an optimization problem, and investigates theoretically properties of its optimal solution. Moreover, it presents a novel automatic technique for solving such a problem. Formally, the proposed algorithm aims at minimizing the summation of the total variations over a given interval of the first derivatives of all the labeled curves, written as functions of a scalar parameter. The algorithm is based on a first-order finite difference approximation of the curves and a sequence of prediction/correction steps. At each step, the predicted points are attributed to the subsequently observed points of the curves by solving an Euclidean bipartite matching subproblem. A comparison with a more computationally expensive dynamic programming technique is presented. The proposed algorithm is applied with success to elastic periodic metamaterials for the realization of high-performance mechanical metafilters. Its output is shown to be in excellent agreement with desirable smoothness and periodicity properties of the metafilter dispersion curves. Possible developments, including those based on machine-learning techniques, are pointed out.

## Introduction

Mechanical metamaterials are artificial materials having properties beyond (“meta”) those of commonly encountered materials^1–4^. They are obtained by assembling multiple elements in repeating patterns, at scales that are smaller than the wavelengths of the physical phenomena of interest. Applications arise, among others, in the design of innovative mechanical filters (called metafilters), which are useful, e.g., in aerospace engineering, bioengineering, and robotics^5–9^.

**Figure 1:**
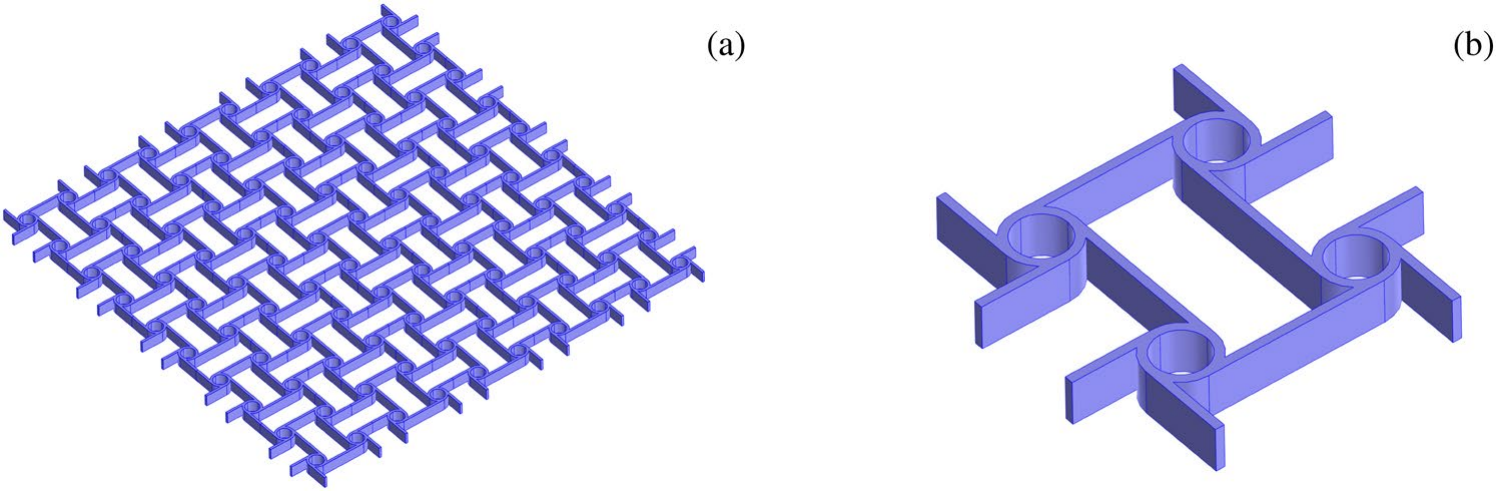
(**a**) A generic portion of an anti-tetrachiral metamaterial. (**b**) Its elementary cell.

**Figure 2:**
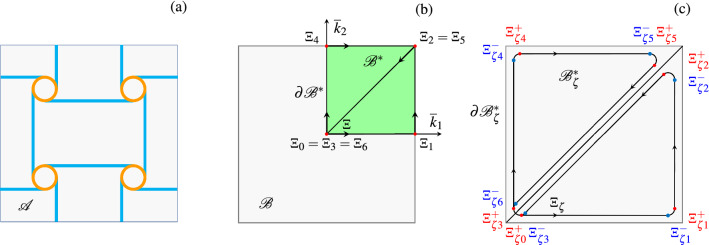
(**a**) Periodic cell $\mathscr {A}$ of the anti-tetrachiral beam-lattice model; (**b**) dimensionless first Brillouin zone $\mathscr {B}\!\!$, first irriducible Brillouin zone $\mathscr {B}\!\!^\star$ and bi-triangular path $\partial \mathscr {B}\!\!^\star$; (**c**) perturbed bi-triangular path, with rounded corners $\partial \mathscr {B}\!\!_{\zeta}^{\star}$ corresponding to the domain $\mathscr {B}\!\!_{\zeta}^{\star}$, parametrized by the perturbation parameter $\zeta >0$.

Current research is often focused on the determination and optimization of dispersion properties of elastic waves in periodic metamaterials, as among others in so-called architected materials which can either function as high-performance advanced phononic crystals^10–15^ or also can host locally resonant devices (periodically or non periodically distributed)^16–21^. Among others, the anti-tetrachiral topology reported in Fig. 1 is an interesting type of metamaterials with high spectral density and markedly auxetic behaviour^22–28^. These metamaterials are obtained by the periodic repetition of an elementary cell, like the one reported in Fig. 1b. In order to reduce the number of variables involved in the mechanical model, it can be convenient resorting to simplified in-plane beam-lattice models where ligaments are modeled as elastic beams and rings as rigid bodies, as shown in Fig. 2a with reference to the periodic cell $\mathscr {A}$ (also known as Wigner–Seitz cell in solid-state physics)^14,25,26^. In order to investigate the dispersion properties in the framework of the Floquet–Bloch theory^29,30^, a periodic reciprocal lattice in the dual space of dimensionless wave vectors $\bar{\textbf{k}} \in \mathbb {R}^2$ is required. The corresponding dual periodic cell $\mathscr {B}\!\!$, i.e., the Wigner–Seitz cell of the reciprocal lattice, is also known as dimensionless first Brillouin zone^31^, schematically reported in Fig. 2b. By exploiting the lattice material symmetries the dimensionless first irreducible Brillouin zone $\mathscr {B}\!\!^\star$ is identified, highlighted in green in Fig. 2b. The frequency spectrum $\bar{\omega }( \bar{\textbf{k}} )$, describing the dispersion properties, is obtained by solving a suitable generalized eigen-problem. In this way, one gets the dimensionless angular eigenfrequency $\bar{\omega }$ parametrized by the wave vector $\bar{\textbf{k}}$ varying along $\partial \mathscr {B}\!\!^\star$ (i.e., the reduced wave vector). In general from an operational point of view, it is necessary to discretize the reduced wave vector and solve repeatedly the eigen-problem. In this way the frequency spectrum, composed of acoustic and optical dispersion curves, is known only on a discrete set of points. To the authors’ knowledge an important open issue consists in attributing each eigenfrequency $\bar{\omega }$ to the corresponding correct (i.e., as smooth as possible) dispersion curve, a problem which may be called *curve* *coloring*. This is addressed in the present paper.

The following are the main contributions of this paper. First, curve coloring is formulated as an optimization problem, and properties of its optimal solution are studied. Second, such an optimization problem is solved by a greedy algorithm, showing its advantages with respect to a more computationally demanding dynamic programming algorithm. Third, smoothness properties of dispersion curves arising in elastic periodic metafilters are proved, as such smoothness is among the assumptions of the curve coloring optimization problem proposed in this paper. Fourth, the proposed greedy algorithm is applied with success to coloring dispersion curves of elastic periodic metafilters, finding automatically their smooth and periodic labeling, which is justified by the ground truth available in this context.

The article is organized as follows: (i) the construction of dispersion curves for periodic metafilters is detailed; (ii) curve coloring is modeled as an optimization problem; (iii) a greedy algorithm is presented for solving it; (iv) smoothness properties of dispersion curves are described, and related to the behavior of that algorithm; (v) results obtained by the curve coloring algorithm are reported, referring to its application to the dispersion curves generated by an existing physical model. The article concludes with a discussion of some possible extensions of this research.

## Dispersion properties for elastic periodic metafilters

**Figure 3:**
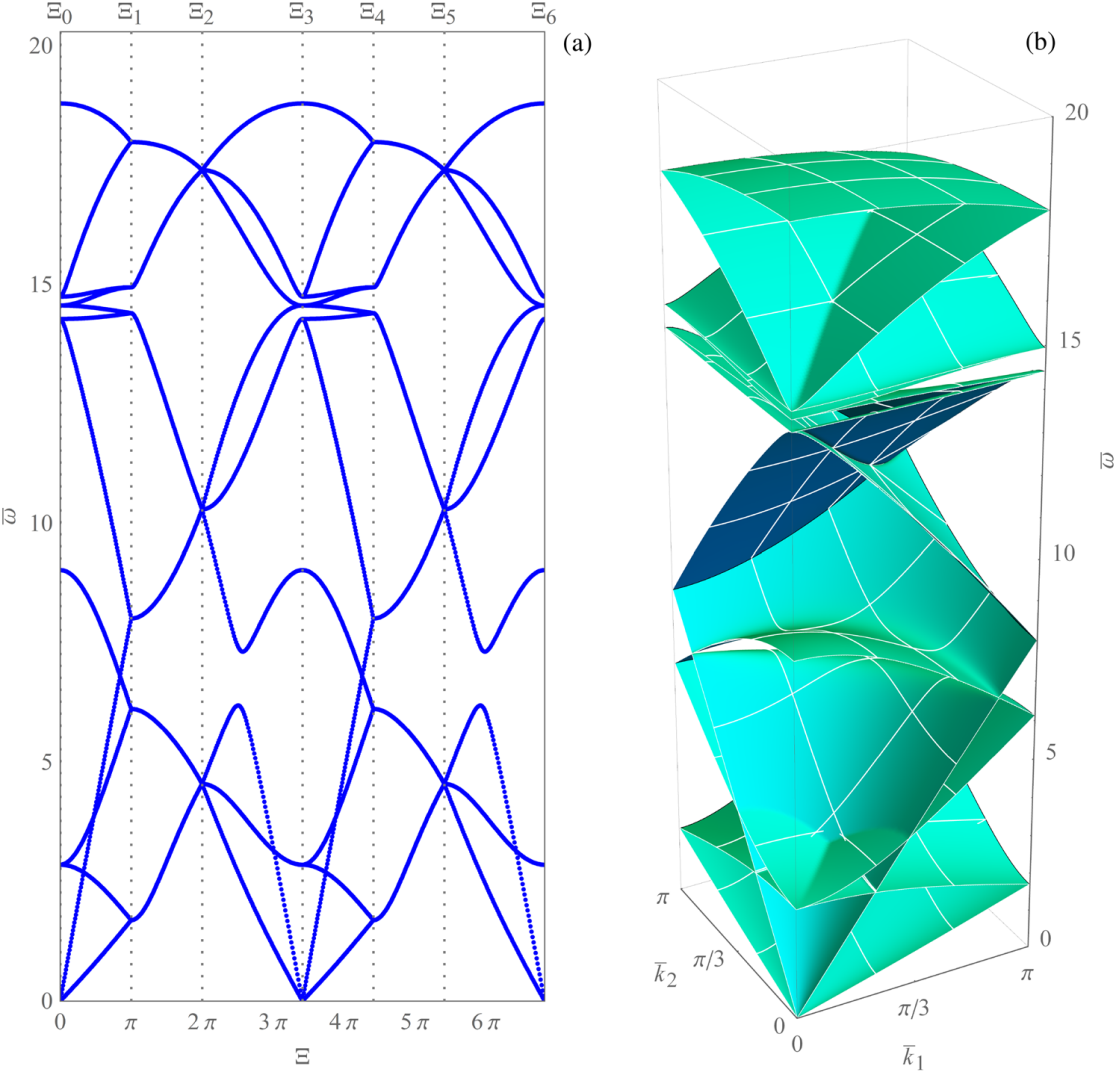
Anti-tetrachiral metamaterial $\mathscr {C}_0$: (**a**) frequency spectrum; (**b**) dispersion surfaces.

In the following, details are given about the determination of the frequency spectrum for an elastic periodic metafilter (e.g., one with anti-tetrachiral topology^25–27^), which can be described via either continuous or discrete Lagrangian models. Let $\bar{\varvec{\mu }} \in \mathbb {R}^p$ denote a dimensionless vector of real design parameters (e.g., geometrical and mechanical parameters) characterizing the metamaterial. By exploiting the Floquet–Bloch theory, the free-wave propagation is investigated by solving the $\bar{\textbf{k}}$-parametrized generalized eigen-problem

1 $$\begin{aligned} \left( \bar{\textbf{K}}\left( \bar{\varvec{\mu }},\bar{\textbf{k}} \right) -\bar{\omega }_h^2\left( \bar{\varvec{\mu }},\bar{\textbf{k}} \right) \bar{\textbf{M}}\left( {{\bar{\varvec{\mu }}}} \right) \right) \bar{\varvec{\psi }}_h \left( \bar{\varvec{\mu }},\bar{\textbf{k}} \right) =\textbf{0}, \end{aligned}$$

where $\bar{\textbf{K}}\left( \bar{\varvec{\mu }},\bar{\textbf{k}} \right) \in \mathbb {C}^{H \times H}$ is the positive semi-definite Hermitian pseudo-stiffness matrix and $\bar{\textbf{M}}\left( {\bar{\varvec{\mu }}} \right) \in \mathbb {R}^{H \times H}$ the positive definite pseudo-mass matrix. In this way, it is possible to determine the generalized eigenvalues $\lambda _h \doteq \bar{\omega }_h^2 \in \mathbb {R}_{\ge 0}$ and the associated generalized eigenvectors $\bar{\varvec{\psi }}_h \in \mathbb {C}^H$ (for $h=1,\ldots ,H$). More specifically, for each choice of $\bar{\varvec{\mu }}$ and $\bar{\textbf{k}}$, both the dimensionless eigenfrequencies $\bar{\omega }_h$ and the corresponding dimensionless polarization vectors $\bar{\varvec{\psi }}_h$ are obtained. By considering the reduced wave vector, i.e., $\bar{\textbf{k}}$ varying on the bi-triangular path $\partial \mathscr {B}\!\!^\star$, for each choice of $\bar{\varvec{\mu }}$, it is possible to determine 2*H* dispersion curves $\bar{\omega }_h({\bar{\textbf{k}}}(\varXi ))$, with $\varXi$ the curvilinear coordinate varying on $\partial \mathscr {B}\!\!^\star$. This is illustrated in Fig. 3a, where due to the symmetry of the curves with respect to the axis $\bar{\omega }=0$, only the *H* curves with $\bar{\omega } \ge 0$ are considered for the anti-tetrachiral metamaterial referred as $\mathscr {C}_0$ in Ref.^26^. Furthermore, in Fig. 3b the dispersion surfaces related to the dimensionless first irreducible Brillouin zone $\mathscr {B}\!\!^\star$ are reported.

As a minor remark it can be observed that the frequency band structure can also be determined by solving an $\bar{\omega }$-parametrized generalized eigen-problem, in terms of auxiliary variables $(\gamma _j)^{\pm 1}=\exp {(\pm \iota \bar{k}_j)}$, with $j=1,2$ and $\iota ^2=- 1$, playing the role of Floquet multipliers and $\bar{k}_j$ being the complex dimensionless wave number. More specifically, for each dimensionless angular frequency $\bar{\omega } \in \mathbb {R}$ and for a given complex multiplier $\gamma _{\alpha }$, with $\alpha =1 \vee 2$, the eigenvalue $\gamma _{\beta ,h} \in \mathbb {C}$, with $\beta =2 \vee 1$ and $\alpha \ne \beta$, as well as the eigenvector $\bar{\varvec{\phi }}_{\beta ,h} \in \mathbb {C}^M$ (for $h=1,\dots ,M$, where *M* is the eigen-problem dimension) are determined. Then, the frequency spectrum $\bar{k}_{\alpha }(\bar{\omega })$ is finally determined exploiting the mapping $\bar{k}_{\beta ,h}=-\iota \ln (\gamma _{\beta ,h})$^32–39^. Based on the obtained frequency spectra, the *relative* *band* *gap* between the *h*-th and *q*-th (consecutive) dispersion functions $\bar{\omega }(\bar{\textbf{k}})$ with $\bar{\textbf{k}} \in \mathscr {B}\!\!^\star$ can be evaluated by solving the $\omega$-parametrized generalized eigen-problem in (1) as

2 $$\begin{aligned} \Delta {{\bar{\omega }}_{h,q, \mathscr {B}\!\!^\star , \text {rel}}}\left( \bar{\varvec{\mu }} \right) =\frac{{\underset{\bar{\textbf{k}}\in \mathscr {B}\!\!^\star }{\min }}{{{\bar{\omega }}}_{q}}(\bar{\varvec{\mu }},\bar{\textbf{k}})-{\underset{\bar{\textbf{k}}\in \mathscr {B}\!\!^\star }{\max }}{{{\bar{\omega }}}_{h}}(\bar{\varvec{\mu }},\bar{\textbf{k}})}{\frac{1}{2}\left[ {\underset{\bar{\textbf{k}}\in \mathscr {B}\!\!^\star }{\min }}{{{\bar{\omega }}}_{q}}(\bar{\varvec{\mu }},\bar{\textbf{k}})+{\underset{\bar{\textbf{k}}\in \mathscr {B}\!\!^\star }{\max }}{{{\bar{\omega }}}_{h}}(\bar{\varvec{\mu }},\bar{\textbf{k}}) \right] }. \end{aligned}$$

It is worth noting that in the case $\bar{\textbf{k}} \in \mathscr {B}\!\!^\star$, Eq. (2) describes full band gaps, while if $\bar{\textbf{k}}$ varies along a given sub-portion of $\partial \mathscr {B}\!\!^\star$, including the origin, partial band gaps can be found. These relative band gaps can be chosen as objective functions of suitable metamaterial optimization problems, as done, e.g., in Refs.^40,41^.

## Curve coloring as an optimization problem

**Figure 4:**
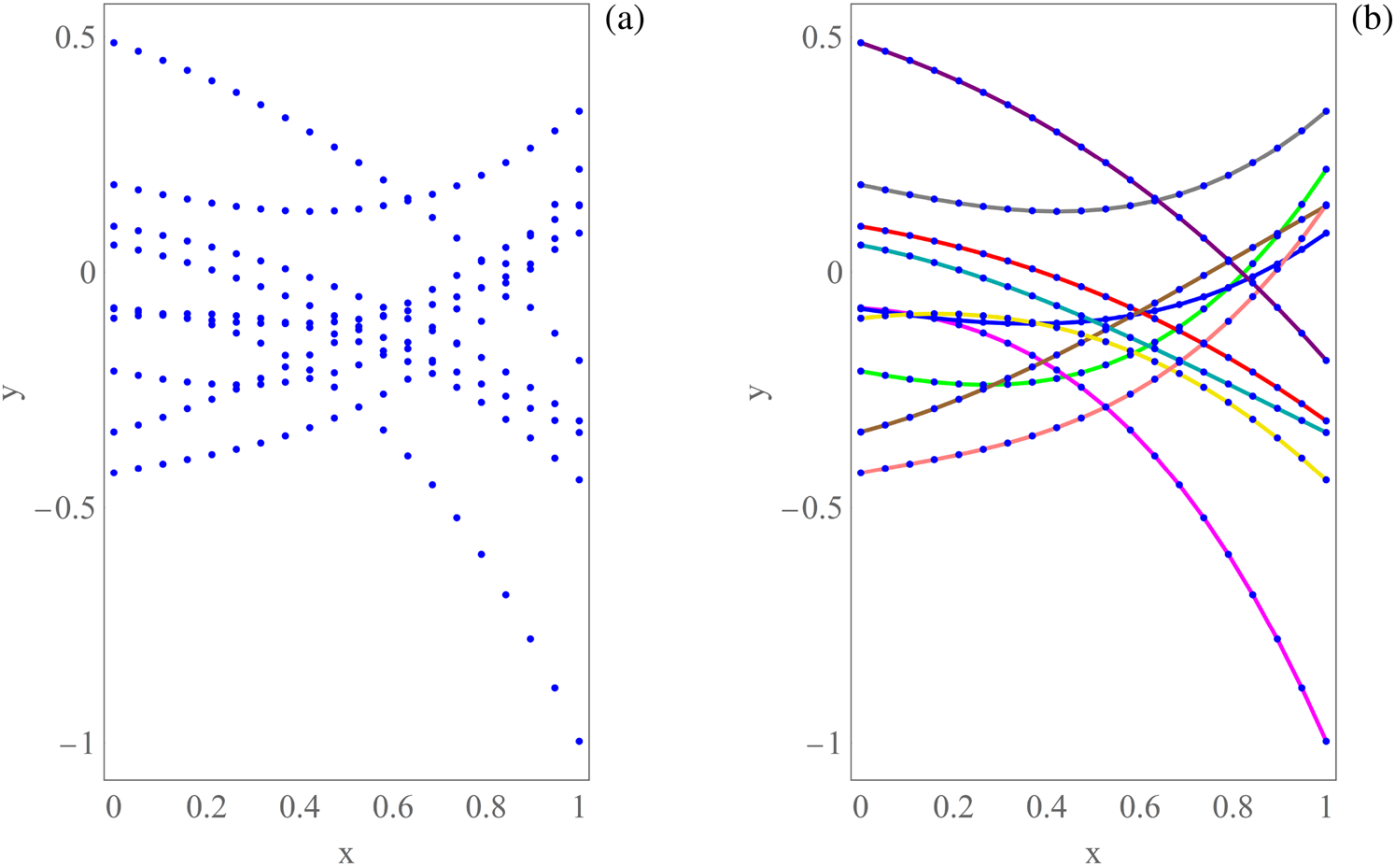
Curve coloring in the case of smooth generic curves. (**a**) Original sampled curves. (**b**) Possible desired output.

For each choice of $\bar{\varvec{\mu }}$, the considered objective function—e.g., a partial relative band gap amplitude—requires the preliminary determination of the dispersion curves $\bar{\omega }_h({\bar{\textbf{k}}}(\varXi ))$ (from now on, the dependence on $\bar{\varvec{\mu }}$ is often omitted in the notation). This can be computationally expensive if a fine discretization grid is used for $\bar{\textbf{k}}$ on the path of interest, because the generalized eigen-problem in Eq. (1) has to be solved for each occurrence of the curvilinear coordinate $\varXi$. As highlighted in Fig. 3, such curves exhibit several crossing and veering phenomena, which calls for their automatic coloring through the development and application of a suitable automatic method, which labels or identifies them appropriately (e.g., maximizing smoothness of the colored curves). This method would take as input several multisets of samples of the curves (without knowing initially to which curve each sample belongs), where each multiset corresponds to a specific value of the independent variable $\varXi$. Its output would be the assignment of each sample to one curve. Figure 4a provides an illustrative example of samples taken from generic smooth curves $y=f_h(x)$, $h=1,\ldots ,H$ with $H=10$, generated as low-degree polynomials with random coefficients without a specific reference to dispersion curves. Moreover, Fig. 4b shows a possible desired (smooth) output of such an automatic method (in this case, based on the ground truth provided by the data-generating process). By the way of example, the figure has been obtained by taking a large spatial sampling frequency and by including a final linear interpolation step after curve coloring. Finding a correct labeling of the curves can help in distinguishing crossings from veerings, being only the latter possibly associated with positive band gaps. Based on the considerations made in this section, curve coloring is presented in the following as an optimization problem, for simplicity referring first to the case of an infinite spatial sampling frequency, then extending the formulation to a finite spatial sampling frequency.

**Figure 5:**
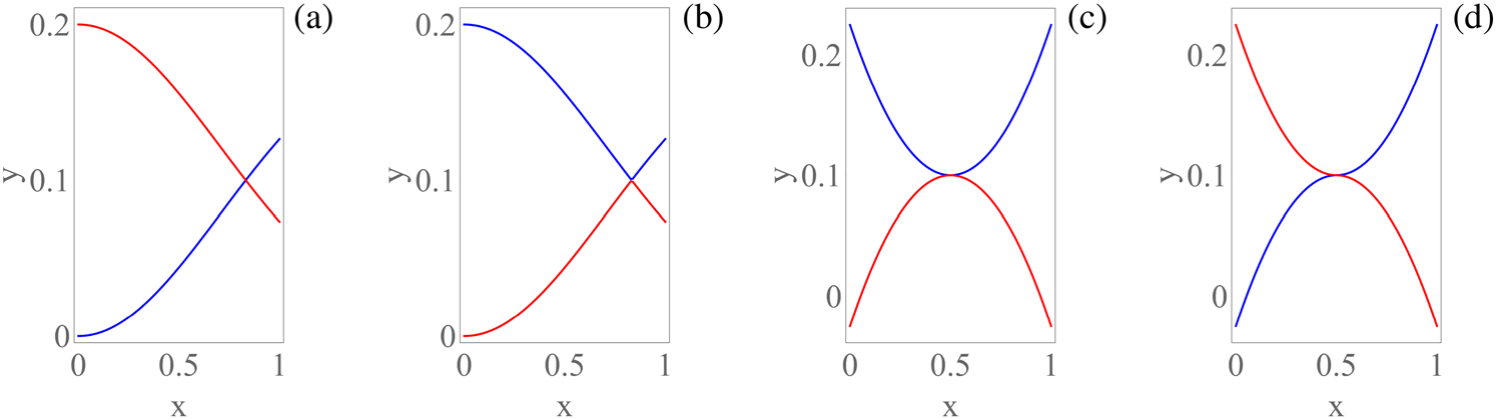
Two curves intersecting at one point, with different slopes: (**a**) curve labeling preserving differentiability at the intersection; (**b**) curve labeling not preserving differentiability at the intersection, and associated with a higher objective value in Eq. (6) than the labeling in part (**a**) of the figure. Two curves intersecting at one point, with the same slope: (**c**) curve labeling preserving differentiability at the intersection; (**d**) nonequivalent curve labeling preserving differentiability at the intersection, and having the same objective value in Eq. (6) as the curve labeling in part (**c**) of the figure.

It is recalled here that, for an interval $[a,b] \subset \mathbb {R}$ and a function $f:[a,b] \rightarrow \mathbb {R}$, the total variation $V_a^b(f)$ of *f* over [*a*, *b*] is defined as

3 $$\begin{aligned} V_a^b(f) \doteq \sup _{P \in \mathscr {P}} \sum _{i=0}^{n_P-1} |f(x_{i+1})-f(x_i)|, \end{aligned}$$

in accordance with Ref.^42^, where the supremum in Eq. (3) is taken over the set of collections of points

4 $$\begin{aligned} \mathscr {P} \doteq&\bigg \{P=\{x_0,\dots ,x_{n_{P}}\}\mid P{\,\,\text{ is }\,\,\text{ a }\,\,\text{ partition }\,\,of\,\,}[a,b] \,\,\text{ satisfying }\,\, x_i < x_{i+1} \,\,\text{ for }\,\, 0\le i\le n_{P}-1\bigg \}. \end{aligned}$$

If $V_a^b(f) < +\infty$, then one says that *f* has bounded variation. In the following, for a positive integer *k*, $\mathscr{P}\mathscr{C}^k([a,b])$ stands for the class of piecewise *k*-times continuously differentiable functions on [*a*, *b*], i.e., whose derivatives up to the order $k-1$ are continuous, whereas the derivative of order *k* is only piecewise continuous (if the latter derivative is also continuous, then one gets the class $\mathscr {C}^k([a,b])$). It is well-known that, if $f \in \mathscr{P}\mathscr{C}^1([a,b])$, then $V_a^b(f)=\int _a^b |f'(x)| dx$. A particularly relevant case in the context of the present paper is when a function can have jump discontinuities. If $f=g+l$ where $g \in \mathscr{P}\mathscr{C}^1([a,b])$ and $l(x)=\sum _{n=1}^N l_n \int _a^x \delta (y-x_n) dy$ (being $\delta$ the Dirac distribution and all the $x_n \in (a,b)$), then

5 $$\begin{aligned} V_a^b(f)=\int _a^b |g'(x)| dx+ \sum _{n=1}^N |l_n|. \end{aligned}$$

The next assumption clarifies the nature of the information available on the unknown curves to be reconstructed. Further details about the assumed smoothness of such curves are given later, with reference to the specific application to periodic metafilters.

### Assumption 1

For $x \in [a,b]$ and *H* (unknown) functions $f_h \in \mathscr{P}\mathscr{C}^2([a,b])$, let the multiset $\bar{F}_x \doteq \{f_h(x) \mid h \in \{1,2,\ldots ,H\}\}$ be given, which provides all the curve observations available for that choice of *x*.

The following assumption clarifies the nature of the admissible set of *H* reconstructed curves.

### Assumption 2

For $h=1,2,\ldots ,H$, let $\hat{f}_h: [a,b] \rightarrow \mathbb {R}$ belong to the class $\mathscr{P}\mathscr{C}^1([a,b])$, and suppose that their first derivatives $\hat{f}_h'$ have the functional form $\hat{f}_h'=g_h+l_h$ where $g_h \in \mathscr{P}\mathscr{C}^1([a,b])$ and $l_h(x)=\sum _{n=1}^N l_{h,n} \int _a^x \delta (y-x_{h,n}) dy$ (being all the $x_{h,n} \in (a,b)$). Finally, assume that, for each $x \in [a,b]$ and each $e \in \bar{F}_x$, there exists a unique index $\bar{h} \in \{1,2,\ldots ,H\}$ for which $\hat{f}_{\bar{h}}(x)=e$ (possibly repeated elements *e*—which are obtained in correspondence of curve intersections—are denoted as different elements of ${\bar{F}_x}$).

Based on Assumptions 1 and 2, curve coloring can be formulated as the following optimization problem. Assumption 2 guarantees a minimal amount of smoothness for the admissible reconstructed curves (which is expressed by the class $\mathscr{P}\mathscr{C}^1([a,b])$), whereas Assumption 1 guarantees that it is possible to label the curves in an even smoother way (which is expressed by the class $\mathscr{P}\mathscr{C}^2([a,b])$).

### Problem 1

Under Assumptions 1 and 2, optimal reconstructed functions $\hat{f}_h^\circ$ ($h=1,\ldots ,H$) are obtained by minimizing the summation of the total variations over [*a*, *b*] of their first derivatives, i.e., by finding

6 $$\begin{aligned} \text{min}_{\{\hat{f}_h \mid h \in \{1,2,\ldots ,H\}\}} \sum _{h=1}^H V_a^b(\hat{f}_h'). \end{aligned}$$

The following proposition motivates the formulation of the optimization problem above. The last part of the proposition refers to the case of periodic curves, which is of interest for the application of curve coloring to dispersion curves arising in the case of periodic metafilters.

### Proposition 1

The following holds:

(i) The optimal solution to Problem 1 has no jump discontinuities in the derivatives of the functions $\hat{f}_h^\circ$.
(ii) If additionally the functions $f_h$ intersect with different derivatives, then the optimal solution is unique (apart from a permutation of the labels) and satisfies $\hat{f}_{\sigma (h)}^\circ =f_h$ for each *h*, where $\sigma$ is a permutation of the set $\{1,\ldots ,H\}$.
(iii) Under the assumptions of item (ii), if the functions $f_{h}$ are periodic with period $b-a$, then the optimal functions $\hat{f}_{h}^\circ$ are also periodic with the same period.

**Proof **Being the functions $\hat{f}_h$ continuous, they coincide (apart from a possible relabeling) with the functions $f_h$ on any interval $\mathscr {I}=[\tilde{a},\tilde{b}]\subseteq [a,b]$ on which they do not intersect. The case of an interval $\mathscr {I}$ in which there is a single intersection at a point $x_0 \in \text{int}(\mathscr {I})$ between two functions $f_1,f_2 \in \mathscr{P}\mathscr{C}^2(\mathscr {I})$, and the two functions intersect with different derivatives at the intersection point, is illustrated in Fig. 5a,b. It is evident that there are only four possible ways of labeling the two curves in that interval (two ways are reported in parts (a) and (b) of the figure, the other two ways are obtained by exchanging the respective colors). In two cases (corresponding to Fig. 5a) there are no jump discontinuities in the first derivatives of the functions $\hat{f}_1$ and $\hat{f}_2$, and one gets $V^{(1)} \doteq \sum _{h=1}^H V_{\tilde{a}}^{\tilde{b}}\left(\hat{f}_h'\right)=\int _{\tilde{a}}^{\tilde{b}} \left( |f_1'(x)|+|f_2'(x)|\right) dx$, whereas in the other two cases (corresponding to Fig. 5b) there are jump discontinuities in the first derivatives of the functions $\hat{f}_1$ and $\hat{f}_2$, and one gets $V^{(2)} \doteq \sum _{h=1}^H V_{\tilde{a}}^{\tilde{b}}\left(\hat{f}_h'\right)=\int _{\tilde{a}}^{\tilde{b}} \left( |f_1'(x)|+|f_2'(x)|\right) dx+\lim _{\varepsilon \rightarrow 0^+} \left( |f_2'(x_0+\varepsilon )-f_1'(x_0-\varepsilon )|+|f_1'(x_0+\varepsilon)-f_2'(x_0-\varepsilon )|\right) >V^{(1)}$. So, the two (equivalent) cases with no jump discontinuities in the first derivatives have lower objective value. However, if $f_1'(x_0)=f_2'(x_0)$, then all the possible (even nonequivalent) labelings of the curves achieve the same objective value when the domain is restricted to $\mathscr {I}$. So, in this case it is not possible to achieve a unique optimal reconstruction of the set of curves, even apart from a permutation of the labels, as illustrated in Fig. 5c,d. The case of an interval containing two or more intersection points and/or the simultaneous intersection of three or more curves is analysed analogously. Concluding, since the case $\hat{f}_h=f_h$ for each *h* provides an admissible labeling of the set of curves, one gets items (i) and (ii) under the respective hypotheses provided in the statement. Finally, item (iii) is an immediate consequence of item (ii) under the additional periodicity assumption on the functions $f_h$.

From a numerical point of view, it emerges that $\bar{F}_x$ is typically observed only on a fine enough grid of points $\{x_s=a+(b-a)s/S \mid s=0,1,\ldots , S=n_P\}$, obtained under a finite spatial sampling frequency $S/(b-a)$ (or equivalently, a finite stepsize $(b-a)/S$). This motivates replacing the generic partition *P* with this $\{x_s\}$ in the definition (3) of total variation and the first derivative $\hat{f}_h'$ with its difference quotient, i.e., the total variation $V_a^b\left(\hat{f}_h'\right)$ over [*a*, *b*] is replaced by its approximation

7 $$\begin{aligned} \bar{V}_a^b\left(\hat{f}_h'\right) \doteq \sum _{i=0}^{S-2} \left| \frac{\hat{f}_h(x_{i+2})-\hat{f}_h(x_{i+1})}{x_{i+2}-x_{i+1}}-\frac{\hat{f}_h(x_{i+1})-\hat{f}_h(x_{i})}{x_{i+1}-x_{i}}\right| . \end{aligned}$$

Then, one gets the following approximate version of Problem 1.

### Problem 2

Under Assumptions 1 and 2, optimal reconstructed functions $\hat{f}_h^\circ$ ($h=1,\ldots ,H$) are obtained by minimizing the summation of the approximations (7), i.e., by finding

8 $$\begin{aligned} \text{min}_{\{\hat{f}_h \mid h \in \{1,2,\ldots ,H\}\}} \sum _{h=1}^H \bar{V}_a^b(\hat{f}_h') = \sum _{i=0}^{S-2} \left( \sum _{h=1}^H \left| \frac{\hat{f}_h(x_{i+2})-\hat{f}_h(x_{i+1})}{x_{i+2}-x_{i+1}}-\frac{\hat{f}_h(x_{i+1})-\hat{f}_h(x_{i})}{x_{i+1}-x_{i}}\right| \right) . \end{aligned}$$

The motivation for Problem 2 is that, under Assumptions 1 and 2 and slight additional smoothness (e.g., if the interval [*a*, *b*] can be partitioned into a finite set of subintervals $\mathscr {I}_i$, and $\hat{f}_h \in \mathscr {C}^2(\mathscr {I}_i)$, for each *h* and *i*), for each admissible set of functions $\hat{f}_h$ ($h=1,2,\ldots ,H$), the approximation $\bar{V}_a^b\left(\hat{f}_h'\right)$ tends to the total variation $V_a^b\left(\hat{f}_h'\right)$ when $S \rightarrow +\infty$.

From a general point of view, Problem 2 can be solved by using Bellman’s equations of dynamic programming^43^, by considering a dynamic optimization problem in which the state of the dynamical system at the stage $i=0, \ldots , S-2$ is provided by an arbitrary matching between the elements of the multisets $\bar{F}_{x_{i}}$ and $\bar{F}_{x_{i+1}}$ (from which one is able to compute the *H*-tuple of difference quotients $\frac{\hat{f}_h(x_{i+1})-\hat{f}_h(x_{i})}{x_{i+1}-x_{i}}$), the control at the same stage is provided by an arbitrary assignment of the elements of the multiset $\bar{F}_{x_{i+2}}$ to the functions $\hat{f}_h$, and the cost-per-stage is $\sum _{h=1}^H\left| \frac{\hat{f}_h(x_{i+2})-\hat{f}_h(x_{i+1})}{x_{i+2}-x_{i+1}}-\frac{\hat{f}_h(x_{i+1})-\hat{f}_h(x_{i})}{x_{i+1}-x_{i}}\right| =\sum _{h=1}^H \left| \frac{\hat{f}_h(x_{i+2})-2\hat{f}_h(x_{i+1})+\hat{f}_h(x_{i})}{\frac{b-a}{S}}\right|$. Nevertheless, for this formulation, the so-called backward phase of dynamic programming (which deals with the construction of suitable functions of the state, called optimal cost-to-go functions, and is more computationally demanding than its forward phase) has computational complexity of order $\mathscr {O}((H!)^2 S)$, since both the numbers of different states and of different controls are equal to *H*!. This is computationally intractable even for small *H* (e.g., for $H = 7$ one gets $(H!)^2 = 25401600$). For this reason, in the following section a greedy algorithm (i.e., based only on a forward phase) is considered, which is shown to produce the same output of the dynamic programming algorithm in examples of technological interest, but with a much smaller computational complexity.

## A greedy algorithm for curve coloring

**Figure 6:**
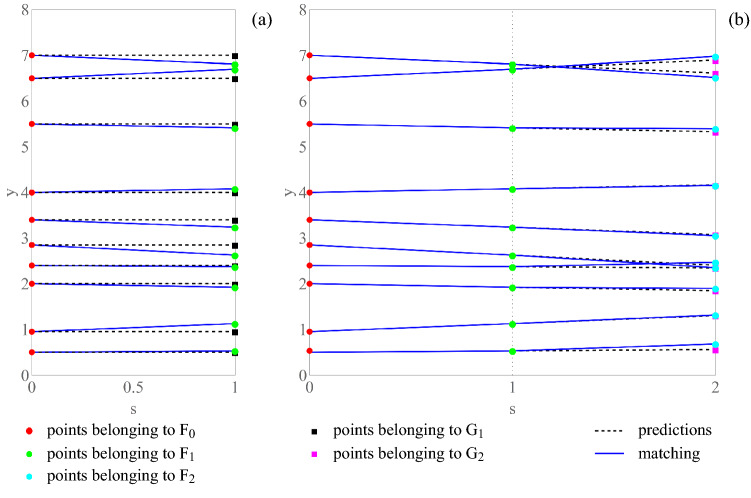
Algorithm 1: (i) First iteration; (ii) Second iteration.

The following greedy algorithm (Algorithm 1) is a generalization of the one proposed in Ref.^44^. The algorithm is based on a first-order finite difference approximation of each curve and on a set of Euclidean bipartite matching subproblems, which are are solved exactly. A backward scheme is used for the first-order finite difference approximation since it relies on information that, at each iteration of the algorithm, is obtained from samples that have been already attributed to each specific curve. The reason for which an Euclidean bipartite matching (sub)problem is solved at each iteration of the algorithm is that the cost function of such a problem is proportional to the summation of the variations of the slopes—from the current value of the abscissa to the successive one—of the curves colored by the algorithm up to that current value of the abscissa. So, this cost function is proportional to one term of the outer summation which appears in the right-most expression (8) of the objective function of Problem 2. Details about the Euclidean bipartite matching problem are deferred to the Supplementary material. It is worth noting that the Euclidean bipartite matching problem has computational complexity of order $\mathscr {O}(H^{2.5} \text{log}(H))$, as detailed in Ref.^45^. Hence, it follows that Algorithm 1 has computational complexity of order $\mathscr {O}(H^{2.5} \text{log}(H) S)$, which is smaller, e.g., than $\mathscr {O}(H^{3} S)$. Specifically, it is smaller than the computational complexity of the dynamic programming algorithm described in the previous section (in the Supplementary material, the two algorithms are compared for a small number *H* of curves, showing that they have the same behavior). To clarify how Algorithm 1 works, in Fig. 6 its first two iterations are illustrated. By construction, Algorithm 1 aims at identifying smooth curves, changing the least possible the slopes of the reconstructed curves from one step to the successive one (this puts it in relation with Problems 1 and 2). For instance, in the case of the example reported in parts (a) and (b) of Fig. 5, Algorithm 1 produces as output the labeling reported in part (a) of the figure (which is associated with the unique optimal solution of each Euclidean bipartite matching subproblem), whereas an alternative simple method, based on the lexicographic order of the curves, produces as output the labeling reported in part (b) of the figure. In the case of the example reported in parts (c) and (d) of Fig. 5, it can be seen that, of the two possible equivalent labelings, Algorithm 1 produces as output the one reported in part (d) of the figure, because of the specific form of its prediction step, based on a backward first-order finite difference approximation of each derivative.

**Table:** 

**Algorithm 1: Curve coloring**
**1:** (Initialization) The curves are evaluated on a regular grid of *S* + 1 values for the curvilinear coordinate *x* on the path of interest (e.g., $\varXi$ on $\partial {\mathscr{B}}^{{ \star }}$) For *s* = 0;1,…, *S* – 1: **2:** (Prediction step) Scanning the curves from the left to the right up to the value *xs* = *a* + (*b* − *a*)*s*/*S* of the curvilinear coordinate *x* on the grid, for each curve one gets a prediction of its point corresponding to *x*_*s*_ + 1, based on a backward first-order finite difference approximation of the curve (the slope of this approximation is equal to 0 for *s* = 0; otherwise, the approximation passes through the points already attributed to the curve in correspondence of *s* and *s* − 1); **3:** (Correction step) The (multi)set *G*_*s*_ + 1 of predicted points is compared with the (multi)set *F*_*s*_ + 1 $\dot{ = }\overline{F}_{{x_{s + 1} }}$ of observed points on the curves, then an Euclidean bipartite matching problem is solved to attribute each observed point to one of the curves, and the corresponding first-order finite difference approximation is updated

### Remark 1

In principle, derivatives could be used in Algorithm 1 instead of difference quotients, but typically at a higher computational cost. For instance, Eq. (10) in the next Remark 4 reports a possible expression of the derivative of each nonzero angular eigenfrequency $\bar{\omega }_h$ with respect to $\varXi$, which is valid, under mild conditions, in the case of the application of the algorithm to metamaterial dispersion curves. Nevertheless, such an expression requires the determination of generalized eigenvectors, whose computational cost increases with their dimension. The Supplementary material shows that the performance of Algorithm 1 is similar to the one would obtain by replacing difference quotients with derivatives.

### Remark 2

In the Supplementary material, Algorithm 1 is also compared with other two algorithms proposed in the literature related to dispersion curve coloring, i.e., in Refs.^46,47^. Although their outputs are generally similar to the one of Algorithm 1, the main advantages of Algorithm 1 are that it can be directly related to the curve coloring optimization problems formulated in the present article (see the next section for a discussion on this issue), and that it does not rely on the computation of generalized eigenvectors, but only of that of generalized eigenvalues. This can be particularly important in high-dimensional problems, characterized by a large number of components of the generalized eigenvectors.

## Smoothness properties of dispersion curves and their relationship with Algorithm 1

In view of the application of Algorithm 1 to the coloring of dispersion curves arising in periodic metafilters, it is important to highlight that in this case one requires a smooth labeling like the one in Fig. 5a, which is produced by Algorithm 1, rather than a non-smooth labeling like the one in Fig. 5b. The reason is that smooth dispersion curves exist under mild conditions, as stated in the next proposition. It is recalled that a nonempty connected open subset of the complex plane is called generically region.

### Proposition 2

The following holds:

(i) Let the positive semi-definite Hermitian matrix $\bar{\textbf{K}}\left( \bar{\varvec{\mu }},\bar{\textbf{k}} \right)$ be a continuously differentiable function of the dimensionless wave vector $\bar{\textbf{k}}$. If $\bar{\textbf{k}}(\varXi )$ is a continuously differentiable function of the curvilinear coordinate $\varXi$ either on the path $\partial \mathscr {B}\!\!^\star$ or on its portion, then it is possible to label each generalized eigenvalue $\bar{\omega }_h^2(\bar{\textbf{k}}(\varXi ))$ in such a way that it is a continuously differentiable function of $\varXi$.
(ii) Let every element of the positive semi-definite Hermitian matrix $\bar{\textbf{K}}\left( \bar{\varvec{\mu }},\bar{\textbf{k}} \right)$ be a holomorphic function of the dimensionless wave vector $\bar{\textbf{k}}$. If each component of $\bar{\textbf{k}}(\varXi )$ is a holomorphic function of the curvilinear coordinate $\varXi$ on a region $\Omega _1$ of the complex plane containing either the path $\partial \mathscr {B}\!\!^\star$ or its portion, then it is possible to label each generalized eigenvalue $\bar{\omega }_h^2(\bar{\textbf{k}}(\varXi ))$ and every component of each generalized eigenvector $\bar{\varvec{\psi }}_h(\bar{\textbf{k}}(\varXi ))$ in such a way that they are holomorphic functions of $\varXi$ on a region $\Omega _2 \subseteq \Omega _1$.

**Proof **By applying the linear mapping $\bar{\varvec{\psi }}_h \mapsto \bar{\textbf{M}}^{1/2} \bar{\varvec{\psi }}_h$ to the generalized eigenvectors $\bar{\varvec{\psi }}_h$ of the generalized eigen-problem reported in Eq. (1), it turns out that such problem can be reduced to a standard eigen-problem, governed by the positive semi-definite Hermitian matrix

9 $$\begin{aligned} \textbf{K}\left( \bar{\varvec{\mu }},\bar{\textbf{k}} (\varXi ) \right) \doteq \bar{\textbf{M}}^{-1/2}\left( \bar{\varvec{\mu }}\right) \bar{\textbf{K}}\left( \bar{\varvec{\mu }},\bar{\textbf{k}} (\varXi ) \right) \bar{\textbf{M}}^{-1/2}\left( \bar{\varvec{\mu }} \right) , \end{aligned}$$

which has the same eigenvalues as the (generalized) eigenvalues of the pair $(\bar{\textbf{K}}\left( \bar{\varvec{\mu }},\bar{\textbf{k}} (\varXi ) \right) ,\bar{\textbf{M}}\left( \bar{\varvec{\mu }}\right) )$. Then, item (i) follows from the application to $\textbf{K}\left( \bar{\varvec{\mu }},\bar{\textbf{k}} (\varXi ) \right)$ of Rellich’s perturbation theorem, stated for the case of continuously differentiable matrices^48^. Similarly, item (ii) follows from the application to $\textbf{K}\left( \bar{\varvec{\mu }},\bar{\textbf{k}} (\varXi ) \right)$ of Rellich’s perturbation theorem, stated for the case of matrices with holomorphic elements^49^.

### Remark 3

Proposition 2(ii) holds under stronger assumptions than Proposition 2(i). However, it provides smoothness not only for the generalized eigenvalues, but also for the generalized eigenvectors. Moreover, it guarantees a higher degree of smoothness than Proposition 2(i). Without the assumptions of Proposition 2(ii), it is easy to construct cases of (generalized) eigen-problems for which the (generalized) eigenvectors are not continuous, even when both the two matrices of the problem and the (generalized) eigenvalues are infinitely differentiable^48^.

### Remark 4

By taking $\bar{\textbf{k}}=\bar{\textbf{k}} (\varXi )$, making the assumptions of Proposition 2(ii), differentiating Eq. (1) as in^40,50–52^, and taking into account that $\bar{\textbf{M}}'=\textbf{0}$, it turns out that the derivative $\bar{\omega }_h'$ of each nonzero angular eigenfrequency $\bar{\omega }_h$ with respect to $\varXi$ has the expression

10 $$\begin{aligned} \bar{\omega }_h'= \frac{\bar{\varvec{\psi }}_h^\star \bar{\textbf{K}}' \bar{\varvec{\psi }}_h}{2 \bar{\omega }_h \bar{\varvec{\psi }}_h^\star \bar{\textbf{M}} \bar{\varvec{\psi }}_h}=\frac{\bar{\varvec{\psi }}_h^\star \bar{\textbf{K}}' \bar{\varvec{\psi }}_h}{2 \bar{\omega }_h}, \end{aligned}$$

where $\bar{\varvec{\psi }}_h^\star$ denotes the complex conjugate transpose of the generalized eigenvector $\bar{\varvec{\psi }}_h$, and the additional normalization $\bar{\varvec{\psi }}_h^\star \bar{\textbf{M}} \bar{\varvec{\psi }}_h=1$ has been made. A similar result holds for the derivative $\lambda _h'=\bar{\varvec{\psi }}_h^\star \bar{\textbf{K}}' \bar{\varvec{\psi }}_h$ of each generalized eigenvalue $\lambda _h=\bar{\omega }_h^2$ with respect to $\varXi$. It follows from Eq. (10) that the curve labeling reported in Fig. 5a is compatible with the continuity of the generalized eigenvectors $\bar{\varvec{\psi }}_h$ (as it produces continuously differentiable nonzero angular eigenfrequencies $\bar{\omega }_h$), whereas the alternative curve labeling reported in Fig. 5b is not compatible with it. Since only two non-equivalent labelings are possible in the particular case shown in Fig. 5a,b, one can conclude that the continuous differentiability of the generalized eigenvalues is equivalent to continuity of the generalized eigenvectors in that situation. This comment can be extended to other situations (e.g., to the case of the simultaneous intersection of three or more dispersion curves, with a different derivative for each function). However, it cannot be extended to the case in which two curves intersect with the same derivative at the intersection point. In this situation, still under the assumptions of Proposition 2(ii), a correct labeling would be obtained as the only one that preserves the continuity of the generalized eigenvectors (at the additional computational cost of determining the generalized eigenvectors, instead of determining only the generalized eigenvalues).

### Remark 5

It is worth highlighting that, in the case of an elastic periodic metamaterial, $\bar{\omega }_h'$ represents the group velocity, i.e., the energy velocity, of elastic waves^50–52^. Hence, one expects the dispersion curves to be Lipschitz continuous. This was proved in Ref.^53^ for the model of periodic metamaterial considered therein.

Concluding, the behavior of Algorithm 1 motivates its application to dispersion curve coloring in the case of elastic periodic metafilters, when the angular eigenfrequencies can be expressed as smooth functions of the curvilinear coordinate $\varXi$ (see Proposition 2 and Remark 4), and the curves intersect with different derivatives at their intersection points (which is a quite common situation in applications). For instance, in the situation described in parts (a) and (b) of Fig. 5, the algorithm is guaranteed to identify these smooth dispersion curves correctly. Furthermore, its output is in agreement also with Proposition 1(i).

It is worth noting that the assumptions of either Proposition 2(i) or Proposition 2(ii) are quite common in models of elastic periodic metafilters. For instance, such models are in general characterized by a pseudo-stiffness matrix $\bar{\textbf{K}}\left( \bar{\varvec{\mu }},\bar{\textbf{k}} \right) \in \mathbb {C}^{H \times H}$ whose elements are holomorphic and single-valued functions of $\bar{\textbf{k}}$, where single-valuedness is also an important property, since it implies periodicity of the dispersion curves on every closed path. Nevertheless, the assumptions of either Proposition 2(i) or Proposition 2(ii) may not hold in the case of the bi-triangular path reported in Fig. 2b, because that path is not a continuously differentiable function of the curvilinear coordinate $\varXi$ (in this case, indeed, one can see from Fig. 3 that the functions $\bar{\omega }_h$ are usually not continuously differentiable in correspondence of the turnaround points of the path $\partial \mathscr {B}\!\!^\star$). Another issue has to do with the fact that in the case of Fig. 3, some curves overlap over a whole interval, which prevents the application of Proposition 1(ii). As shown by the numerical results reported in the next section, however, both issues can be solved by considering a slight perturbation $\partial \mathscr {B}\!\!_{\zeta}^{\star}$ (with rounded corners) of the bi-triangular path, which is shown later in Fig. 2c. The perturbed path is constructed by joining 6 segments and 6 circular arcs (2 with the same center). The parameter $\zeta >0$ represents both the distance of each segment from the corresponding one in the original path, and the radius of each circumference. It is worth mentioning that, for the beam-lattice metamaterial class of models considered, the assumptions of Proposition 2(i) hold for the perturbed path (so the nonzero dispersion curves are continuously differentiable), whereas the assumptions of Proposition 2(ii) hold for each portion of the perturbed path (e.g., the segments, the circular arcs) that can be represented as the graph of a holomorphic function. Hence, Assumption 1 holds for all the nonzero dispersion curves (i.e., it may not hold only for dispersion curves that are equal to 0 locally). It is also worth mentioning that Remark 4 suggests, as an alternative, to apply Algorithm 1 not directly to the dispersion curves, but to the generalized eigenvalue curves $\bar{\omega }_h^2({\bar{\textbf{k}}}(\varXi ))$, then to transfer the resulting labels from the reconstructed generalized eigenvalue curves to the corresponding dispersion curves.

Finally, in the Supplementary material, an alternative use of Algorithm 1 (i.e., its application to generalized eigenvector curves instead of dispersion curves) is briefly outlined, showing that the original application is preferable according to various points of view.

## Results

**Figure 7:**
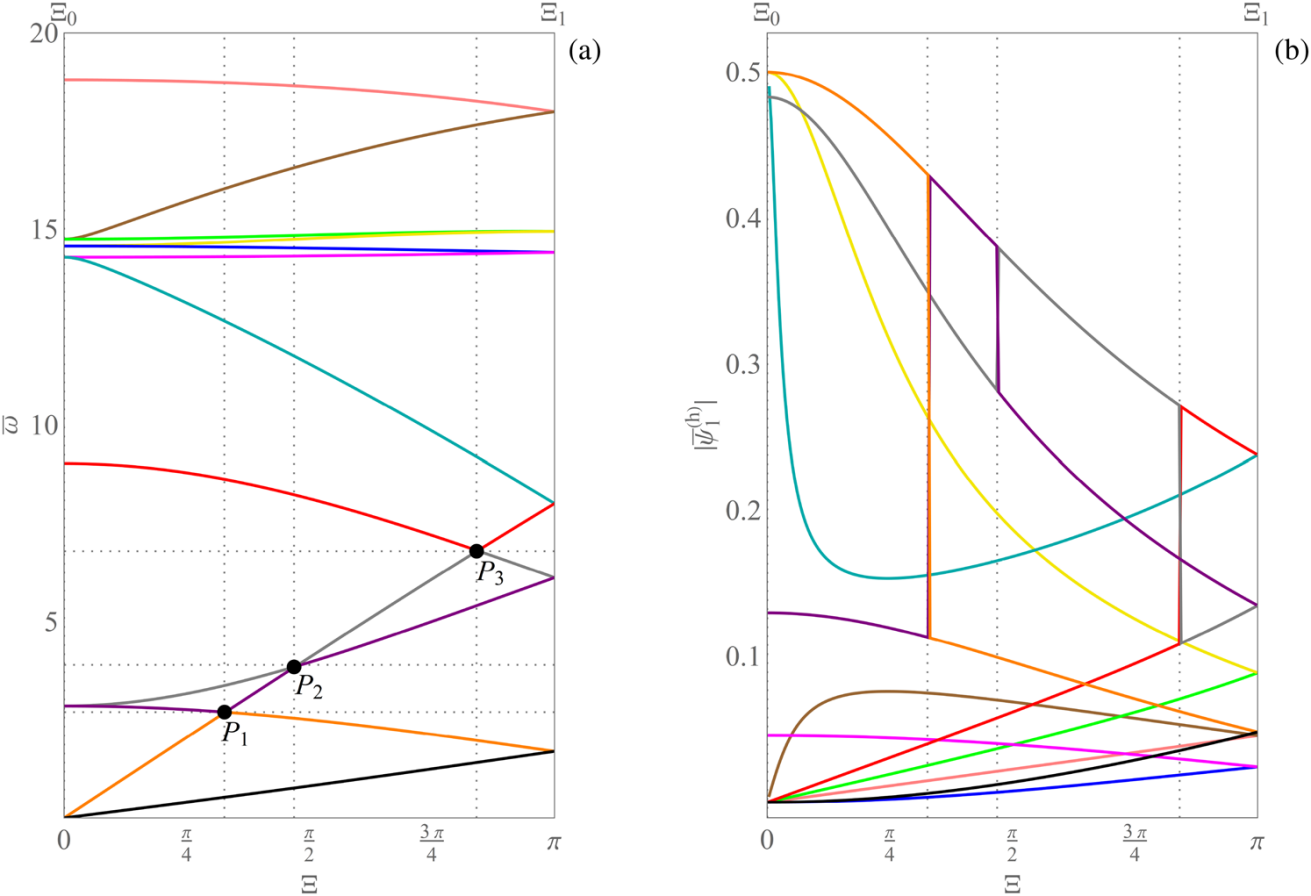
(**a**) Dispersion curve coloring obtained by the alternative simple method. (**b**) Loss of continuity of the magnitude components $|\bar{\psi }_1^{(h)}|$ corresponding to the generalized eigenvector curves, labeled in accordance with the results reported in part (**a**) of the figure.

This section is devoted to the curve coloring of frequency spectra $\bar{\omega }(\bar{\textbf{k}})$ corresponding to the beam-lattice metamaterial referred as $\mathscr {C}_0$ in Ref.^26^, by exploiting the proposed Algorithm 1. Note that this algorithm can be successfully used for the curve coloring of generic elastic architectured metamaterials described by either continuous or discrete Lagrangian models. In order to better highlight the performance of Algorithm 1, its results are compared with the corresponding ones obtained by the alternative simple method, based on the lexicographic order of the dispersion curves. For the sake of convenience, the stepsize has been chosen large enough to be able to discriminate between crossings (true intersections) and veerings (no intersections). More precisely, the stepsize has been chosen to be equal to $\pi /100$, which corresponds to $S=100$ in Fig. 7 (the robustness of the numerical results with respect to the choice of the stepsize is investigated in the Supplementary material). Furthermore, for the cases examined, the same results have been also obtained by applying Algorithm 1 to the generalized eigenvalue curves, then transferring the labels to the corresponding dispersion curves.

**Figure 8:**
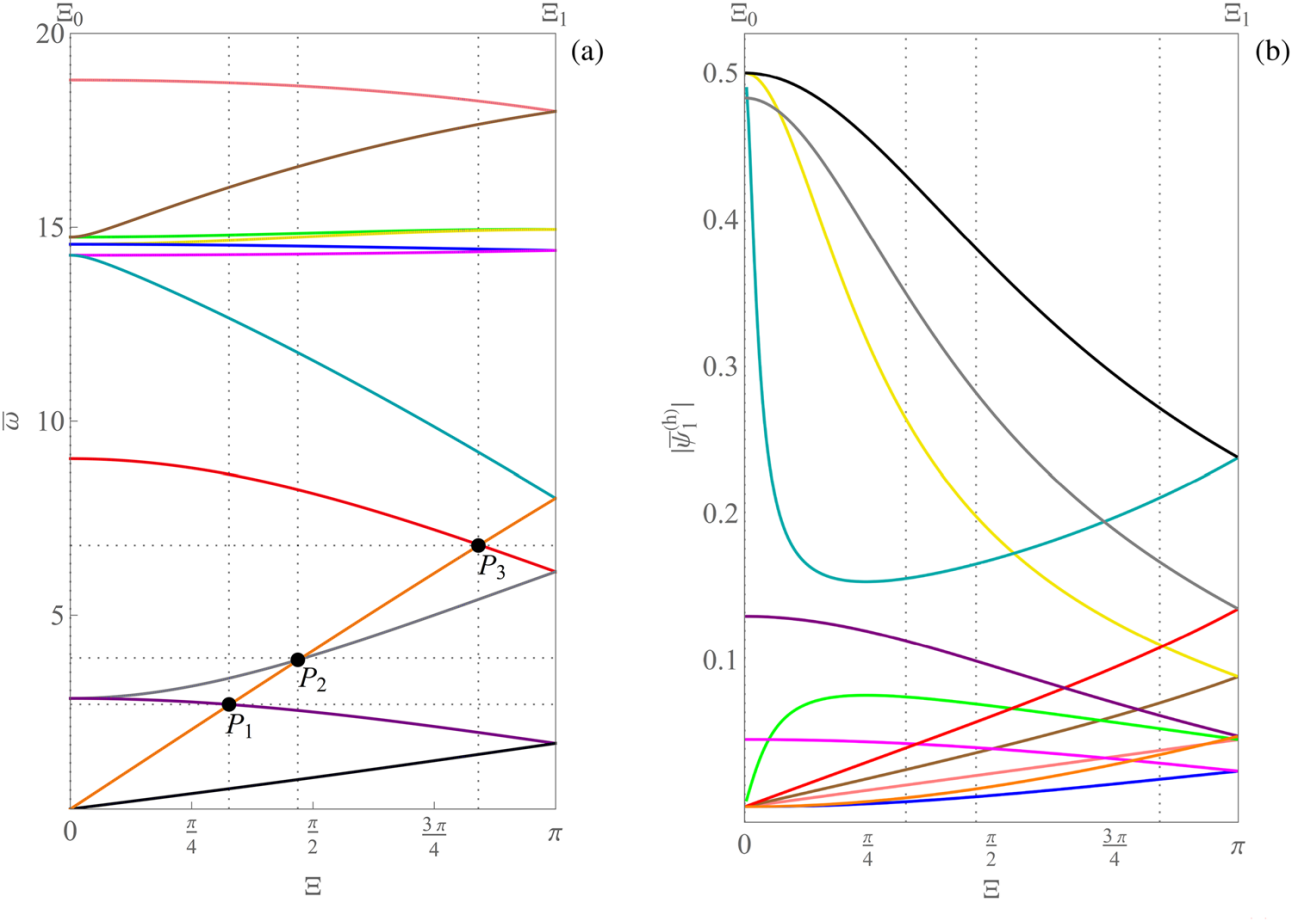
(**a**) Dispersion curve coloring obtained by Algorithm 1. (**b**) Continuity of the magnitude components $|\bar{\psi }_1^{(h)}|$ corresponding to the generalized eigenvector curves, labeled in accordance with the results reported in part (**a**) of the figure.

**Figure 9:**
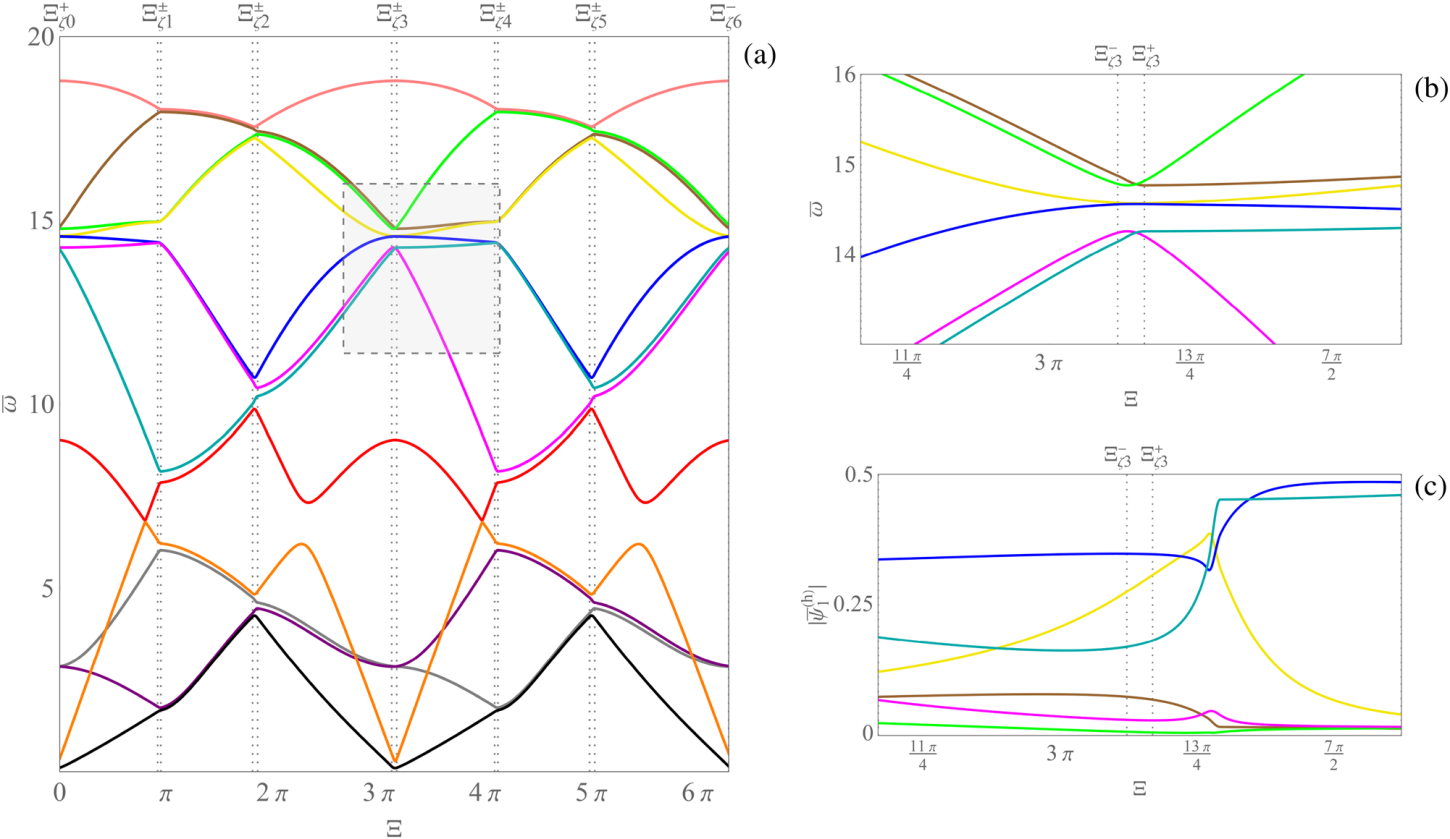
(**a**) Dispersion curve coloring obtained by Algorithm 1, in the case of the perturbed bi-triangular path; (**b**) zoomed view of the gray shaded area in part (**a**) of the figure; (**c**) magnitude components $|\bar{\psi }_1^{(h)}|$ of the generalized eigenvector curves corresponding to the zoomed view shown in part (**b**) of the figure.

Figure 7a shows the set of dispersion curves reconstructed by the simple method, on the path $\varXi _0$-$\varXi _1$ of Fig. 2b. It is evident that in points $P_1$, $P_2$, $P_3$ some branches show loss of differentiability. Moreover, the Fig. 7b highlights the loss of continuity of the magnitude components $|\bar{\psi }_1^{(h)}|$ corresponding to the generalized eigenvector curves. The generalized eigenvectors have been normalized to have unit norm. Similar results are obtained for the other magnitude components of the generalized eigenvectors. It follows that the curve coloring obtained via the simple method does not provide smooth curves as in Proposition 2 and, therefore, cannot be a valid tool for the correct labeling of dispersion curves. On the other hand, Fig. 8a shows the set of dispersion curves reconstructed by Algorithm 1, on the path $\varXi _0$-$\varXi _1$ of Fig. 2b. In this case, as expected, no loss of differentiability of the reconstructed curves is observed as it emerges by considering points $P_1$, $P_2$, $P_3$. Figure 8b shows the associated labeling of the magnitude components $|\bar{\psi }_1^{(h)}|$ corresponding to the generalized eigenvectors. The figure highlights the continuity of such curves. Differently from the previous case, the obtained curve coloring is in accordance with the smoothness provided by Proposition 2(ii). Indeed, being every element of the matrix $\bar{\textbf{K}}\left( \bar{\varvec{\mu }},\bar{\textbf{k}} \right)$ a holomorphic function of $\bar{\textbf{k}}$, and being each component of $\bar{\textbf{k}}(\varXi )$ a holomorphic function of $\varXi$, it turns out that it is possible to label the nonzero dispersion curves and the generalized eigenvalues in such a way that they are holomorphic functions of $\varXi$. Finally, Fig. 9a shows the results obtained by Algorithm 1 on the perturbed bi-triangular path $\partial \mathscr {B}\!\!_{\zeta}^{\star}$, which is shown in Fig. 2c. Figure 9b illustrates a zoomed view of the dispersion curves, in the gray shaded area reported in part (a) of the figure. Then, Fig. 9c highlights the smoothness of the magnitude components $|\bar{\psi }_1^{(h)}|$ corresponding to the generalized eigenvector curves, labeled in accordance with the results reported in part (a) of the figure. It is evident from Fig. 9 that: the perturbation of the path makes the dispersion curves smoother with respect to Fig. 3a (which are evaluated on the original bi-triangular path $\partial \mathscr {B}\!\!^\star$) and separates curves that in Fig. 3a overlap over a whole interval; moreover, in this case, Algorithm 1 reconstructs smooth and periodic curves, as expected. It is evident that Algorithm 1 can be effectively used for the curve coloring of dispersion spectra. To conclude, it has to be remarked that, when the periodic dispersion surfaces are continuous functions of the dimensionless wave vector $\bar{\textbf{k}}$ on the dimensionless first irreducible Brillouin zone $\mathscr {B}\!\!^\star$ (which occurs under mild conditions^54^, which are satisfied in the present context for the case of either item (i) or (ii) of Proposition 2), then they are also uniformly continuous on any compact set containing $\mathscr {B}\!\!^\star$. This guarantees that, for any $\varepsilon >0$, it is possible to choose the value of the perturbation parameter $\zeta$ in such a way that the global maximum/minimum of each dispersion curve moves at most by $\varepsilon$ as a consequence of the perturbation, guaranteeing that each computed absolute band gap is perturbed at most by $2\varepsilon$ in absolute value. Analogously, perturbation bounds can be found for computed relative band gaps.

## Conclusion and future developments

In this paper, curve coloring has been formalized as an optimization problem, and properties of its optimal solution have been proved. Then, a greedy algorithm has been considered for its numerical resolution. The relationship of the algorithm with the proposed curve coloring optimization problem and its approximate version has been discussed, by finding an agreement between theoretical properties of the optimal solution to such problem and the solution found by the algorithm. The algorithm itself has been motivated by the existence of a smooth labeling of the generalized eigenvalue curves, which is among the assumptions of the proposed curve coloring optimization problem. Such smoothness has been theoretically investigated in the context of dispersion curves arising in elastic periodic metafilters. Finally, the algorithm has been successfully applied for the coloring of such dispersion curves. Following the same arguments provided here, it can be proved that the proposed algorithm can be successfully exploited for the curve coloring of periodic materials with generic topology described by discrete Lagrangian or discretized continuous models, provided that the wave propagation is studied via an eigen-problem satisfying the properties required by the Rellich’s perturbation theorem, and that the dispersion curves intersect with different derivatives at their intersection points. More specifically, under the stated conditions, it can be observed that the proposed curve coloring algorithm can be successfully used to identify correctly the branches of the frequency band structures in linear elastic, piezoelectric, magneto-electroelastic materials, among others, as long as dissipative phenomena do not occur. Compared with other algorithms proposed in the literature^46,47^, the main advantages of the proposed algorithm are that it can be directly related to the curve coloring optimization problems formulated in the present article, and that it does not rely on the computation of generalized eigenvectors, but only of that of generalized eigenvalues.

In the following, some possible future directions of the present research are discussed. First, by possibly making additional assumptions, the relationship between Problems 1 and 2 could be further investigated. Furthermore, it is worth mentioning that Proposition 2 does not easily extend to the two-dimensional case (i.e., to the case of dispersion surfaces, for what concerns the application to periodic metafilters) because it is well-known that Rellich’s perturbation theorem holds only for the one-dimensional case, i.e., for eigenvalues expressed in terms of a single scalar variable^48^. Moreover, that theorem cannot be applied when the angular eigenfrequencies are complex (i.e., in the case of periodic metafilters with dissipation). Indeed, there exist several theoretical examples in the literature in which the generalized eigenvalues are not smooth functions of a parameter, even when the two matrices that appear in the generalized eigen-problem are smooth functions of that parameter (see, e.g.,^55^). However, dispersion curves/surfaces obtained for several models of metamaterials are quite smooth, even in the presence of dissipation (see, e.g.,^56^), so a suitable variation of Rellich’s perturbation theorem may hold for these cases. On the other hand, an additional extension concerns the possibility of modifying Algorithm 1 by adopting in it a second-order finite difference approximation of the reconstructed curves instead of a first-order finite difference approximation. In this way, in the situation described in parts (c) and (d) of Fig. 5, the modified algorithm is expected to produce the labeling illustrated by part (c) of the figure. This would correspond to replacing first derivatives with second derivatives in the objectives of Problems 1 and 2. A further enhancement regards the possible improvement of the dynamic programming algorithm discussed in the present paper, by taking into account additional properties of the dispersion curves, such as Lipschitz continuity, which was proved in Ref.^41^ for certain models of periodic metafilters. However, for applications of technological interest, estimates of the Lipschitz constants would be needed. Another possible extension regards the combination of Algorithm 1 with machine learning methods, e.g., with the aim of achieving an adaptive sampling of the set of dispersion curves, or of automatically determining a stepsize able to distinguish crossings from veerings (taking as training set a collection of labeled images representing sets of sampled curves). Finally, the algorithm could be applied to the case of infinite-dimensional generalized eigen-problems arising in continuum metafilter models, by focusing on a portion of the frequency spectrum containing a finite number of dispersion curves.

## Supplementary Information

Supplementary Information.

## Data Availability

The datasets used and/or analysed during the current study are available from the corresponding author on reasonable request.
