# Supplementary material for: On dispersion curve coloring for mechanical metafilters
Source: Sci Rep. 2022 Nov 21;12:20019. doi: 10.1038/s41598-022-23491-4 (PMC9681881; doi:10.1038/s41598-022-23491-4)
Supplement: Supplementary file 1 — Supplementary Information. [file 41598_2022_23491_MOESM1_ESM.pdf]

Supplementary material of

On dispersion curve coloring for mechanical metafilters

Andrea Bacigalupo, Maria Laura De Bellis, Giorgio Gnecco, Federico Nutarelli

Supplementary Section 1: Euclidean bipartite matching problem

The next concepts from graph theory are recalled here (see also Ref.[1] for details). Let $G = (V, E, d)$ be a weighted undirected graph with vertex set V, edge set E, and the weight function $d : E \to \mathbb{R}$, where $d(u, v)$ represents the weight of the edge $(u, v) \in E$. A matching $M \subseteq E$ is a subset of edges such that every vertex in V is incident to at most one edge in M. The matching is perfect if every vertex in V is incident to exactly one edge in M. The cost of the matching has the expression $c \doteq \sum_{(u,v)\in M} d(u, v)$. The Euclidean bipartite matching problem is formulated as follows.

Problem 1. *Given a first set R of n red points and a second set B of n blue points in the plane, let $G = (R \cup B, E, d)$ be the complete weighted bipartite graph with the property that there is an edge between any two points if and only if they are of different colors. Additionally, for each $u \in R$ and $v \in B$, the weight $d(u, v)$ of the edge (u, v) is expressed by the Euclidean distance between the points u and v. The objective is to find a perfect matching in this graph having minimum cost c.*

For illustrative purposes, Supplementary Figure 1(a) reports the optimal solution to a two-dimensional version of the Euclidean bipartite matching problem. However, it is worth remarking that in Figure 6(b), its one-dimensional version is considered. Then, Supplementary Figure 1(b) highlights the worst such solution, i.e., the one corresponding to the largest cost. Finally, Supplementary Figure 1(c) reports the costs of all possible matchings.

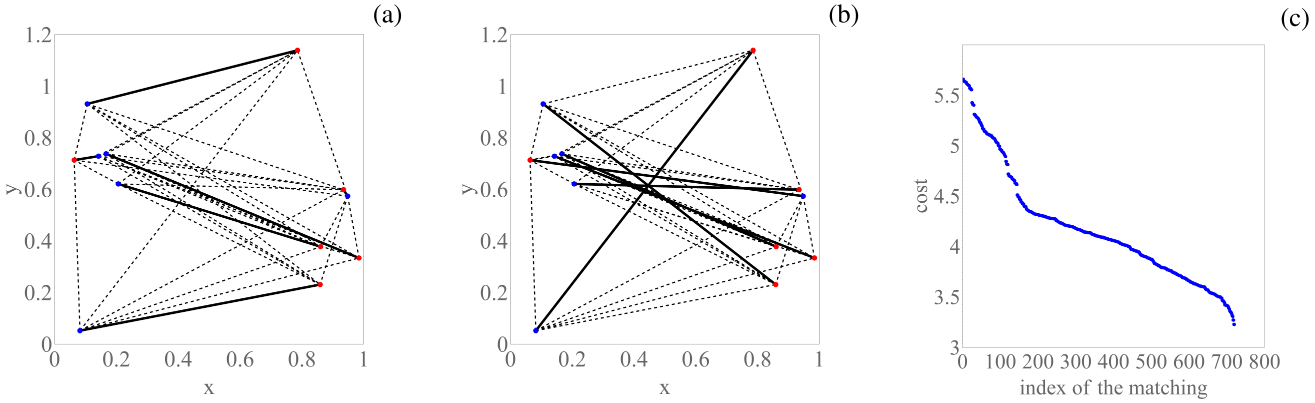

Supplementary Figure 1. (a) Optimal solution to a two-dimensional version of the Euclidean bipartite matching problem. The edges corresponding to that matching are reported in bold. (b) Corresponding worst solution (i.e., having the largest cost c). The edges corresponding to that matching are reported in bold. (c) Cost of each matching. The costs are reported from the largest one (associated with part (b) of the figure) to the smallest one (associated with part (a) of the figure).

Supplementary Section 2: Stepsize choice

Supplementary Figure 2 shows numerically that the behavior of Algorithm 1 does not depend significantly on the choice of the stepsize, at least for a quite broad interval of values for it. In general, a small stepsize would guarantee a good accuracy of the approximation of the derivative with the difference quotient, and also of a linear approximation of the curve (based either on the derivative or on the difference quotient). Still, it would be associated with a large number of iterations of the algorithm, hence with a large total computational cost. The opposite would occur in the case of a large stepsize, for which the approximations above may be poor, but the total computational cost would be small. Nevertheless, it is worth observing that, thanks to the alternation of prediction and correction steps and the inclusion of Euclidean bipartite matching subproblems, the algorithm can label the curves correctly, even in the presence of possibly poor approximations of the curves in correspondence of the successive sample, as illustrated in Supplementary Figure 3.

Supplementary Remark 1. *More generally, it is possible to investigate the issue above analytically as follows, for simplicity limiting to the case of a single intersection of two functions $f_1, f_2 \in \mathscr{PC}^2(\mathscr{I})$, having different derivatives $f_1'(x^\star)$ and $f_2'(x^\star)$ at their intersection point $x^\star$. Let the iteration number s be such that $x^\star \in [x_s, x_{s+1})$, and, without loss of generality, assume*

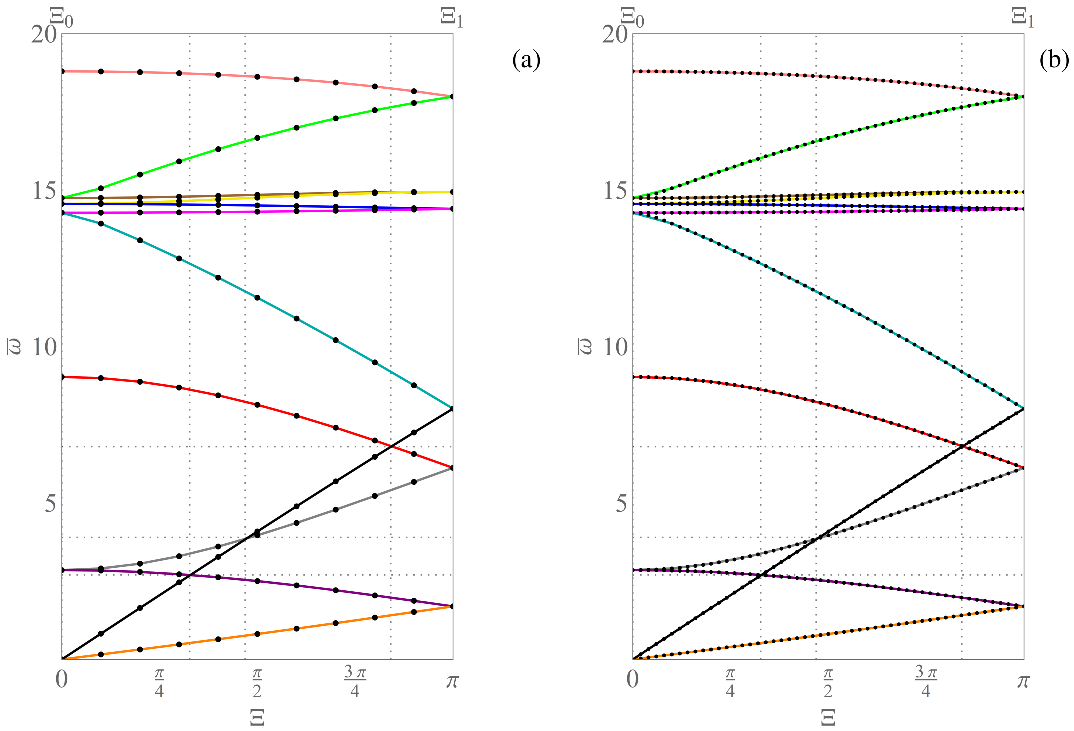

Supplementary Figure 2. Dispersion curve coloring obtained by Algorithm 1 (the locations of the original unlabeled samples are highlighted in black). (a) Stepsize $\pi/10$. (b) Stepsize $\pi/50$. Similar results are obtained for the stepsizes $\pi/100$ (considered in the main text) and $\pi/1000$ (not reported here due to graphical reasons).

$f_1(x_s) \leq f_2(x_s)$ and $f_1'(x^\star) > f_2'(x^\star)$ (see again Supplementary Figure 3 for a visualization of this situation, with f_1 and f_2 being the functions highlighted in blue and red, respectively). Moreover, suppose that the stepsize $(b-a)/S$ is small enough in such a way that, for some $\varepsilon > 0$, one has $f_1'(x^\star) - \varepsilon > f_2'(x^\star) + \varepsilon$, $f_1'(x) \in [f_1'(x^\star) - \varepsilon, f_1'(x^\star) + \varepsilon]$ and $f_2'(x) \in [f_2'(x^\star) - \varepsilon, f_2'(x^\star) + \varepsilon]$ for all $x \in [x_{s-1}, x_{s+1}]$. Then, due to Lagrange's mean value theorem, both the backward difference quotient $\frac{f_1(x_s) - f_1(x_{s-1})}{x_s - x_{s-1}}$ and the derivative $f_1'(x_s)$ belong to $[f_1'(x^\star) - \varepsilon, f_1'(x^\star) + \varepsilon]$, and similarly, both the backward difference quotient $\frac{f_2(x_s) - f_2(x_{s-1})}{x_s - x_{s-1}}$ and the derivative $f_2'(x_s)$ belong to $[f_2'(x^\star) - \varepsilon, f_2'(x^\star) + \varepsilon]$. Now, in the prediction step, the algorithm would produce the estimates

$$\begin{aligned} f_1(x_{s+1}) &\simeq \hat{f}_1(x_{s+1}) \\ &= f_1(x_s) + \frac{f_1(x_s) - f_1(x_{s-1})}{x_s - x_{s-1}}(x_{s+1} - x_s) \\ &\geq f_1(x_s) + (f_1'(x^\star) - \varepsilon)\frac{b-a}{S} \\ &\geq f_1(x^\star) - (f_1'(x^\star) + \varepsilon)(x^\star - x_s) + (f_1'(x^\star) - \varepsilon)\frac{b-a}{S} \\ &\geq f_1(x^\star) + f_1'(x^\star)(x_{s+1} - x^\star) - 2\varepsilon\frac{b-a}{S}, \end{aligned} \tag{S1}$$

and

$$\begin{aligned} f_2(x_{s+1}) &\simeq \hat{f}_2(x_{s+1}) \\ &= f_2(x_s) + \frac{f_2(x_s) - f_2(x_{s-1})}{x_s - x_{s-1}}(x_{s+1} - x_s) \\ &\leq f_2(x_s) + (f_2'(x^\star) + \varepsilon)\frac{b-a}{S} \\ &\leq f_2(x^\star) - (f_2'(x^\star) - \varepsilon)(x^\star - x_s) + (f_2'(x^\star) + \varepsilon)\frac{b-a}{S} \\ &\leq f_2(x^\star) + f_2'(x^\star)(x_{s+1} - x^\star) + 2\varepsilon\frac{b-a}{S}. \end{aligned} \tag{S2}$$

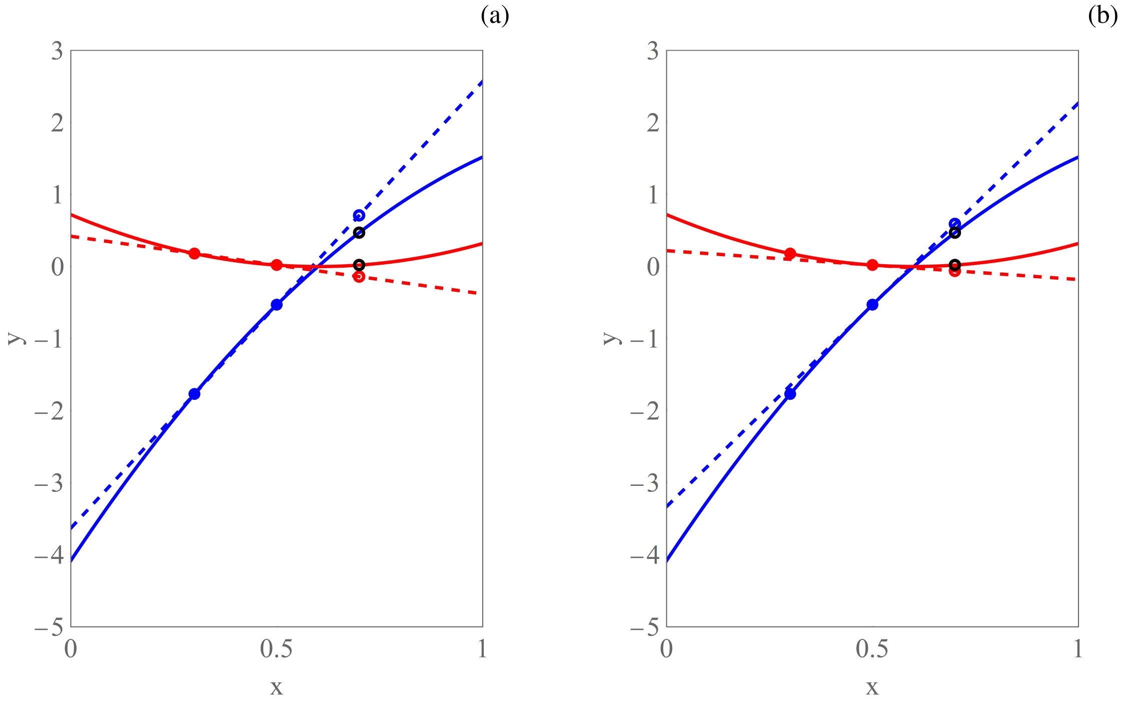

Supplementary Figure 3. Curve coloring for $x = 0.7$, given the current point $x = 0.5$, and the previous point $x = 0.3$ (the locations of the samples that have still to be labeled at that iteration are highlighted by unfilled circles in black, whereas the locations of the samples that have already been labeled at that iteration are highlighted with filled red or blue circles). (a) Predictions for $x = 0.7$ (indicated by unfilled red and blue circles) obtained by Algorithm 1 before its correction step. (b) Predictions for $x = 0.7$ (indicated by unfilled red and blue circles) obtained before the correction step by a variation of Algorithm 1 in which the difference quotients at $x = 0.5$ are replaced by derivatives at $x = 0.5$. In both cases, the final labeling obtained by solving the Euclidean bipartite matching subproblem in the correction step is consistent with the ground truth.

Since $f_1(x^\star) = f_2(x^\star)$, the bounds above guarantee

$$\hat{f}_1(x_{s+1}) > \hat{f}_2(x_{s+1}) \tag{S3}$$

for

$$(f_1'(x^\star) - f_2'(x^\star))(x_{s+1} - x^\star) > 4\varepsilon \frac{b-a}{S}\,. \tag{S4}$$

In this case, the correction step confirms the choice made in the prediction step, and the algorithm attributes the points to the correct curves. The condition provided by Supplementary Eq. (S4) is likely to occur since $f_1'(x^\star) > f_2'(x^\star)$, $x^\star$ is expected to be located anywhere on $[x_s, x_{s+1})$, and ε goes to 0 as $(b-a)/S$ goes to 0. Still, it is interesting to observe that, even in the case in which that condition is not satisfied because, e.g., $x_{s+1} - x^\star$ is too small compared to $(b-a)/S$, the algorithm is still able to locate approximately the intersection and reconstruct correctly the curves before and after the intersection, as illustrated by Supplementary Figure 4.

It is also worth mentioning that a too large stepsize could make it impossible to detect crossings, as illustrated in Supplementary Figure 5. Such an issue would be not associated with the use of the specific Algorithm 1, but actually with its input. In other words, any other algorithm based on that resolution of the curves is expected to miss crossings. One possible way to solve this issue and be reasonably confident that a crossing has not been missed by Algorithm 1 is to apply it with various decreasing stepsizes, checking if its behaviour does not change with the stepsize (likewise in Supplementary Figure 2).

A final remark has to do with the use of an adaptive stepsize instead of a fixed one in Algorithm 1, in order to reduce its number of iterations, without decreasing its accuracy in the reconstruction of the curves. One possibility could be to increase the stepsize when, for a certain number of consecutive iterations, the coloring produced by the algorithm is the same as the one produced by the alternative simple method, based on the lexicographic order of the curves, and the curves are sufficiently far from each other (according to the current estimates of their derivatives, and the current choice of the stepsize). In the opposite case, the stepsize would be reduced. One could also keep track of the iterations in which the coloring produced by Algorithm

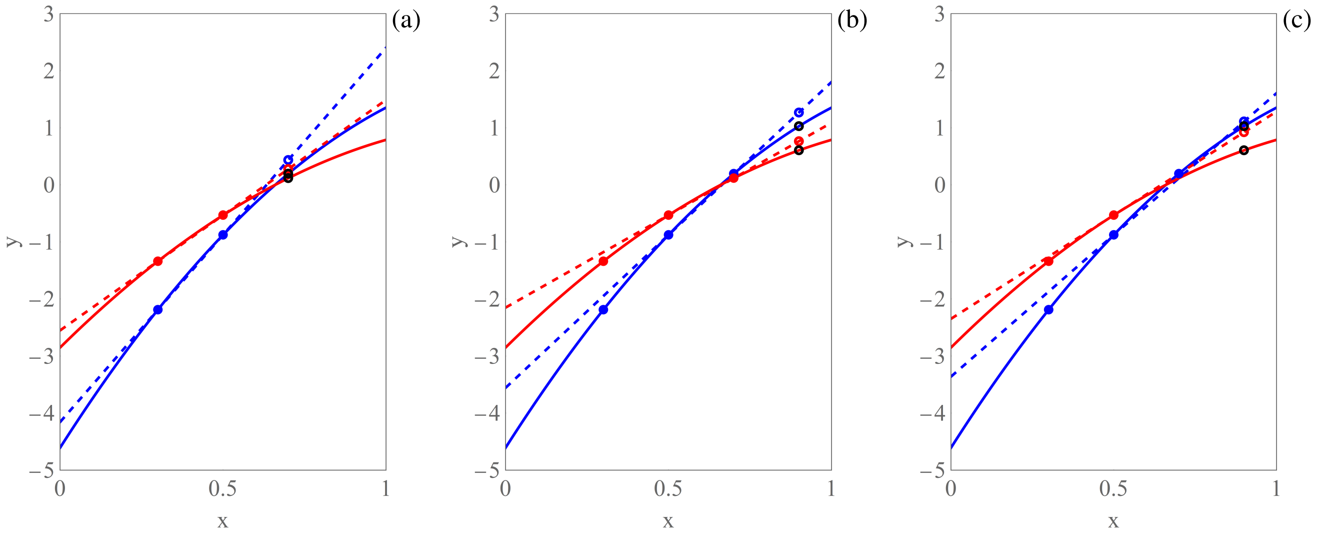

Supplementary Figure 4. Curve colorings (the locations of the samples that have still to be labeled at that iteration are highlighted by unfilled circles in black, whereas the locations of the samples that have already been labeled at that iteration are highlighted with filled red or blue circles). At each iteration, predictions obtained before the correction step are indicated by unfilled red and blue circles. (a) Curve coloring for $x = 0.7$, given the current point $x = 0.5$, and the previous point $x = 0.3$. Since the Euclidean bipartite matching subproblem has two optimal solutions, two possible equivalent labelings are obtained, which are considered in the two successive subfigures. (b) Curve coloring for $x = 0.9$, given the current point $x = 0.7$, the previous point $x = 0.5$, and one optimal solution of the previous Euclidean bipartite matching subproblem. (c) Curve coloring for $x = 0.9$, given the current point $x = 0.7$, the previous point $x = 0.5$, and the other optimal solution of the previous Euclidean bipartite matching subproblem. In both cases (b) and (c), the obtained labeling agrees with the ground truth for $x = 0.5$ and $x = 0.9$, although a labeling that does not agree with the ground truth could have been obtained for $x = 0.7$, see case (c).

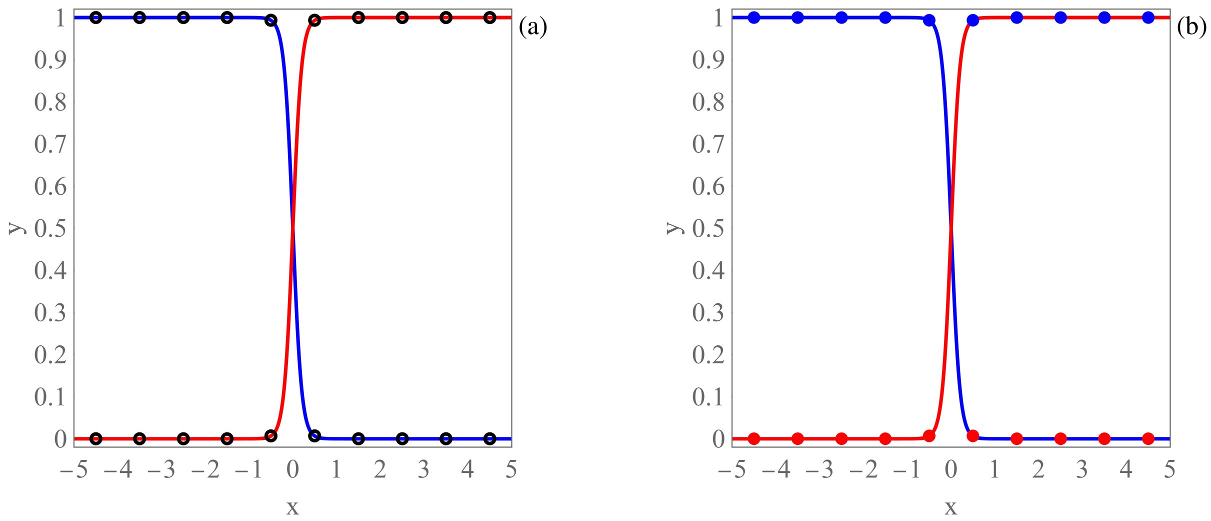

Supplementary Figure 5. Curve coloring for the case of a too coarse grid. (a) Original ground truth curves and locations of the original unlabeled samples, highlighted by unfilled circles in black. (b) Curve coloring produced by Algorithm 1 (and by any other reasonable algorithm based on the available samples).

1 differs from the one produced by the alternative simple method, in order to further refine the stepsize therein, for a possible second application of the algorithm, made for checking purposes. A detailed investigation of these issues is a possible topic of future research. It is worth keeping in mind, however, that the inclusion of an adaptive stepsize would impact the complexity of the algorithm, due to the additional computational burden required to update the stepsize according to a suitable rule.

Supplementary Section 3: Application of Algorithm 1 to generalized eigenvector curves

In this section, the alternative application of Algorithm 1 to generalized eigenvector curves is briefly outlined. In this case, each such curve lies on a higher-dimensional space than the corresponding dispersion curve. Since there are no intersections of generalized eigenvector curves, at a first sight, in this alternative case the application of Algorithm 1 would be easier. However, this alternative approach is actually not straightforward, due to the following reasons.

- Solving the Euclidean bipartite matching subproblems is computationally more expensive with respect to the original application of the algorithm, due to the larger dimension of the space.
- Even in the simplest case in which each generalized eigenvalue has multiplicity 1, unit-norm generalized eigenvector curves are defined within a multiplicative complex scalar, which may depend on a parameter (e.g., on the curvilinear coordinate Ξ) when a general-purpose algorithm is applied to find numerically the generalized eigenvectors.

The last issue can be partly solved in the following case, in which one specific component (e.g., the first one) of all the generalized eigenvectors found numerically by changing the curvilinear coordinate, is always different from 0. In this case, by assuming that there exist continuous generalized eigenvector curves, one can pre-process the data by making each such component equal to a positive real number, possibly depending on the curvilinear coordinate. This is simply obtained by multiplying each numerically found generalized eigenvector by a suitable unit-norm complex number, making it possible to remove the phase from that component. With this pre-processing, Algorithm 1 has been applied to the set of generalized eigenvector curves, in the case of the same beam-lattice model considered in the present article (and also in the case of the other models investigated in Ref.[2]). Then, dispersion curves have been labeled in the same way as the labeled generalized eigenvector curves. The results found in this way have turned out to be the same as the one reported in Figures 8 and 9, but have required a larger computational effort to be obtained.

Supplementary Section 4: Comparison of Algorithm 1 with other algorithms for curve coloring

First, Algorithm 1 is compared with the dynamic programming algorithm briefly presented in the main text, for a simple artificial example having $H = 3$ curves and $S = 20$. In this case, both the numbers of different states and of different controls are equal to $H! = 6$, and one gets $(H!)^2 = 36$, so the application of dynamic programming is still computationally tractable. Supplementary Figure 6 shows that, in this situation, there is agreement of the results obtained by the two algorithms. In the specific example, the ground truth has been obtained by generating 3 intersecting polynomials of degree 3, with random coefficients. Similar results have been obtained for other choices of the random coefficients. It is worth remarking that, by well-known properties[3], the dynamic programming algorithm achieves an optimal solution of Problem 2.

Second, Algorithm 1 is compared with two algorithms for dispersion curve coloring reported in the literature, namely, in Refs.[4,5]. An important difference is that, in the analysis reported in the presented article, the behavior of Algorithm 1 is related to Problem 2, whereas in the references above the behavior of the two alternative algorithms is not analyzed with reference to an optimization problem whose objective expresses the smoothness of the colored curves. The main ideas of the two alternative algorithms are summarized as follows.

The algorithm proposed in Ref.[4] is based on the combination of two local approximations for each curve (namely, a linear approximation and a quadratic one), which are used to define a search region for the next sampled point to be attributed to that curve. Moreover, the stepsize of the algorithm is chosen in an adaptive way, based on a suitable parameter. At each iteration, sampled points already attributed to a curve are removed from the current set of unlabeled sample points. As a check for correctness, in the case of search regions containing several still unlabeled sampled points, suitable information on the (generalized) eigenvectors is taken into account. An inspection of the numerical results reported in Figure 8 of that reference shows that its curve labeling is not related to solving Problem 2 (i.e., it does not necessarily minimize the approximation of the summation of the total variations of the slopes of the labeled curves), differently from Algorithm 1. This is likely due to the fact that, at each iteration s, sampled points in the multiset F_{s+1} are attributed to curves sequentially, whereas Algorithm 1 assigns simultaneously such points to all the curves, by solving the related Euclidean bipartite matching subproblem. Moreover, looking at the (generalized) eigenvectors has a cost which increases with their dimension, whereas Algorithm 1 does not need them when all the intersections occur with different derivatives for the intersecting curves. Also constructing local quadratic approximations requires an additional computational cost with respect to the case in which only local linear approximations

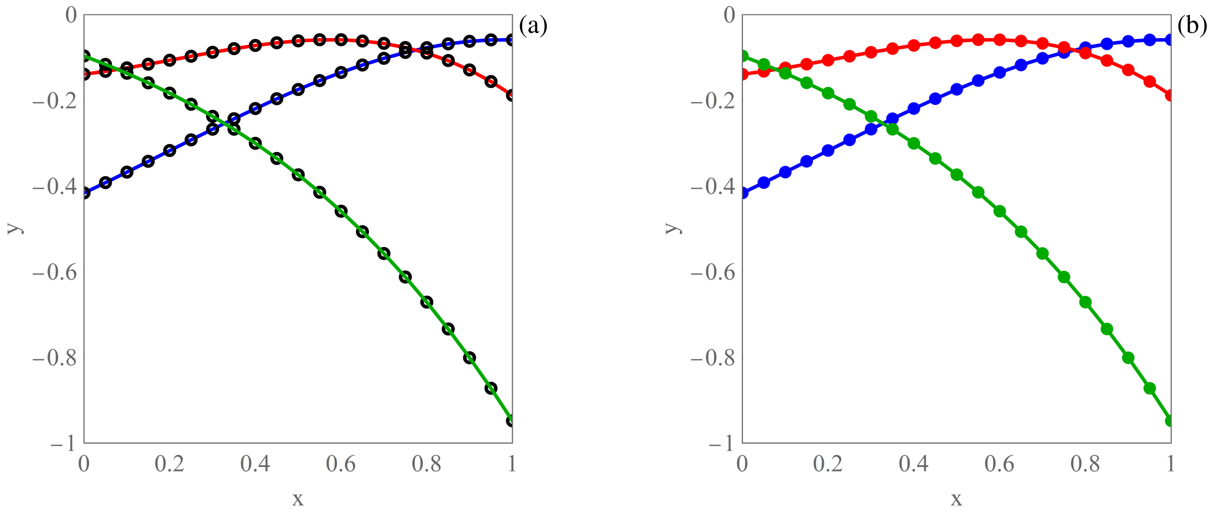

Supplementary Figure 6. Curve coloring for the case of $H = 3$ curves and $S = 20$. (a) Original ground truth curves and locations of the original unlabeled samples, highlighted by unfilled circles in black. (b) Same coloring produced by both Algorithm 1 and the dynamic programming algorithm.

are used.
Similarly, also the algorithm proposed in Ref.[5] works by looking at the set of (generalized) eigenvectors, in this case by imposing their mutual orthogonality at each iteration (according to a suitable inner product, in case of generalized eigenvectors). As such, it produces essentially the same output as Algorithm 1 in the cases (already discussed in the main text) in which the dispersion curves intersect with different derivatives at their intersection points. Still, that algorithm requires information about the (generalized) eigenvectors, whose computational cost increases with the dimension, as already discussed for the other alternative algorithm.
Finally, another difference between Algorithm 1 and the two alternative algorithms above is that it can be applied also in the case in which the curves to be colored are not associated with a parametrized (generalized) eigen-problem. Such situations may arise, e.g., in applications to image processing.

References

1. Vaidya, P. M. Geometry helps in matching. *SIAM J. on Comput.* **18**, 1201–1225 (1989).
2. Bacigalupo, A., Gnecco, G., Lepidi, M. & Gambarotta, L. Optimal design of low-frequency band gaps in anti-tetrachiral lattice meta-materials. *Compos. Part B: Eng.* **115**, 341–359 (2017).
3. Bertsekas, D. *Dynamic programming and optimal control*, vol. 1 (Athena Scientific, 2012).
4. Bause, F., Rautenber, J. & Henning, B. An improved mode-tracing algorithm to compute dispersion curves of acoustic waveguides. In *Proceedings of the 2010 IEEE International Ultrasonics Symposium*, 719–722 (2010).
5. Houillon, L., Ichchou, M. N. & Jezequel, L. Wave motion in thin-walled structures. *J. Sound Vib.* **281**, 483–507 (2005).
